# Perfectionism and psychological well-being in adolescents with high intellectual abilities

**DOI:** 10.3389/fpsyg.2025.1617755

**Published:** 2025-07-22

**Authors:** M. Rosa Sánchez-Moncayo, Inmaculada Menacho, Pedro Ramiro, José I. Navarro

**Affiliations:** ^1^Junta de Andalucia, Cadiz, Spain; ^2^Department of Psychology, University of Cadiz, Cadiz, Spain

**Keywords:** perfectionism, high intellectual abilities, well-being, adolescence, education, giftedness

## Abstract

**Introduction:**

Perfectionism is understood as a psychological construct that can either facilitate excellence in various areas of life or lead to maladaptation when not properly managed. Although numerous studies exist on perfectionism and high abilities, this relationship still has many unresolved scientific questions, especially regarding the emotional development of individuals.

**Method:**

This study aimed to analyze the relationship between perfectionism and psychological well-being in 103 adolescents aged 12 to 17 years with either high intellectual abilities or average intellectual capacity. Subgroups were established based on different age ranges to compare the relationships between perfectionism and psychological well-being throughout adolescence.

**Results:**

The results showed statistically significant differences in the mean levels of perfectionism according to the subjects’ intellectual abilities, with the mean being higher in the high intellectual ability groups. However, there was no statistically significant correlation between perfectionism and psychological well-being in any of the groups, regardless of intellectual capacity or age. Although adaptive perfectionism was more common in adolescents with high abilities, this did not necessarily translate into higher levels of psychological well-being.

**Discussion and conclusion:**

The study highlights the complex relationship between high intellectual ability (HIA) and perfectionism, distinguishing between adaptive and maladaptive forms. While HIA adolescents show higher perfectionism levels, their psychological well-being remains comparable to peers. Findings emphasize the role of educational and cultural contexts, urging tailored interventions to foster adaptive perfectionism and mitigate its negative effects.

## Introduction

Research on perfectionism has evolved considerably over the past decades, transitioning from early unidimensional models that predominantly linked perfectionism to negative outcomes, to more sophisticated, multidimensional frameworks that differentiate between adaptive and maladaptive features (Fletcher et al., 2023; Pákozdy et al., 2023; Smith et al., 2022). Early investigations primarily associated perfectionism with detrimental effects such as low self-esteem, heightened anxiety, and emotional vulnerability (Plucker and Callahan, 2014; Zhao et al., 2024). However, more recent studies have redefined perfectionism as a form of cognitive control that can be both beneficial and harmful, according to its expression. In this view, perfectionism encompasses at least two distinct components: the imposition of high-performance standards and the presence of concerns regarding mistakes (Fletcher et al., 2023; Rinn, 2024).

This comprehensive viewpoint is particularly pertinent when examining individuals with High Intellectual Abilities (HIA) (Fernández-Mera et al., 2024). Historically, HIA populations have been stereotypically viewed as predisposed to mental health challenges and psychosocial imbalances, often attributed to maladaptive perfectionistic tendencies (Baudson and Preckel, 2016; Kuznetsova et al., 2024; Mofield and Parker, 2015). However, most contemporary researchers adopt a more standardized perspective, recognizing the multidimensional nature of perfectionism. They understand it as a continuum of behaviors and thoughts, ranging from positive aspects—associated with achievement, excellence, and well-being (i.e., healthy perfectionism)—to negative or maladaptive forms that hinder success and undermine personal well-being (e.g., Stoeber, 2018). Recent research, has begun to challenge these assumptions: Vicent et al. (2019) and Sastre et al. (2019) have illustrated that the advanced metacognitive skills commonly observed in HIA individuals may facilitate a more adaptive management of perfectionism, fostering high achievement without the associated psychological distress. Moreover, longitudinal studies (e.g., Endleman et al., 2022) indicate that the evolution of perfectionistic tendencies across developmental stages calls for a deeper understanding of how these traits interact with intellectual capacity over time.

Emerging evidence also highlights the moderating influence of contextual and cultural factors on perfectionism. For instance, research by Chan (2008) and Fong and Yuen (2014) shows that Eastern cultural norms, which valorize strategies for managing social differences, may promote adaptive perfectionism, in contrast to the more conformity-driven paradigms often observed in Western contexts. These cultural distinctions underscore the importance of examining perfectionism within a broader socio-cultural framework, particularly among adolescents with HIA, who may experience unique social pressures and academic expectations (Rodríguez, 2025; Neihart, 2021; Senol et al., 2023).

Other studies (Sand et al., 2021; Mofield and Parker, 2015) have explored how various dimensions of perfectionism affect the psychological well-being of adolescents with high intellectual abilities (HIA). Perfectionism is often categorized into two primary dimensions: perfectionistic strivings, which involve setting high personal standards, and perfectionistic concerns, characterized by excessive worry over mistakes and fear of negative evaluation. Research indicates that perfectionistic concerns are linked to negative psychological outcomes, such as depression and social anxiety, among gifted adolescents. Alternatively, perfectionistic strivings can correlate with positive outcomes, including higher achievement and well-being, when not accompanied by significant concerns over mistakes. This suggests that the impact of perfectionism on psychological well-being in HIA adolescents is nuanced, with the potential for both beneficial and detrimental effects depending on the specific dimensions and their interplay, although significant open questions still exist regarding the impact of perfectionism during adolescence, particularly in adolescents with high intellectual abilities.

In addition to the aforementioned advances, research has focused on the underlying mechanisms contributing to perfectionistic tendencies. Environmental influences such as parental expectations and academic pressures significantly contribute to the development of both adaptive and maladaptive forms of perfectionism (Damian et al., 2013; Noor, 2023). Their findings indicate that the perfectionism observed in HIA individuals is not solely an innate trait but is also shaped by external factors and early educational experiences. Interpersonal relationships and peer comparisons play a critical role in reinforcing perfectionistic behavior during adolescence—a period marked by rapid cognitive and emotional development (Saß et al., 2025). Moreover, neuropsychological studies have begun to identify distinct neural activation patterns associated with high performance standards and error sensitivity, suggesting a biological substrate that differentiates adaptive from maladaptive perfectionism (Beljan et al., 2024).

Alongside, Grugan et al. (2021) emphasized the importance of considering socio-cultural influences in shaping perfectionism, revealing that cultural norms and societal expectations modulate perfectionistic behaviors. Complementing these findings, Ogurlu (2020) proposed an integrative framework that encompasses both intrinsic cognitive factors and extrinsic environmental determinants, providing a more holistic understanding of perfectionism in HIA populations. These studies collectively underscore the complexity of perfectionism and highlight the need for targeted interventions that address both individual and contextual variables.

### Objectives of the current study

Taking into account the connections between perfectionism and HIA this study seeks to address several key questions:

1. What is the relationship between intellectual capacity (HIA vs. Average Intellectual Capacity, AIC) and types of perfectionism (adaptive vs. maladaptive)?
2. How do different dimensions of perfectionism influence psychological well-being among HIA adolescents?
3. What role do contextual factors (e.g., cultural background, educational environment) play in moderating these relationships?

By investigating these questions, our aim was to refine theoretical models of perfectionism and its interplay with high intellectual ability, thereby informing targeted interventions that leverage adaptive cognitive control strategies while mitigating the detrimental effects of maladaptive perfectionism.

## Method

### Participants

A total of 103 students aged between 12 and 17 years participated in this study. The overall mean age was 14.2 (SD = 1.4). Participants were divided into two groups: (1) adolescents with High Intellectual Ability (HIA) (*n* = 52); and (2) adolescents with Average Intellectual Capacity (AIC) (*n* = 51). The HIA group had a mean age of 14.3 (SD = 1.41) and the AIC group had a mean age of 14.1 (SD = 1.31). Regarding gender, the groups were not homogeneous due to the incidental sample: 53.4% were girls, with girls being less frequent in the HIA group compared to the AIC group (42.3% vs. 64.7%). Each of the groups was further subdivided into two subgroups based on age. The differentiation of subgroups by age range followed developmental criteria, resulting in four subgroups: (1) HIA Group 1, aged 12 to 14 years; (2) HIA Group 2, aged 15 to 17 years; (3) AIC Group 1, aged 12 to 14 years; and (4) AIC Group 2, aged 15 to 17 years. The groups were within the same age range (12–17 years), with equivalent means (14.3 and 14.1 years for HIA and AIC respectively) (see Table 1). All participants were young students residing in different municipalities with populations exceeding 50,000 inhabitants. The sample selection was based on incidental criteria. The sole exclusion criterion for participating in any group was a prior diagnosis of developmental disorder or psychopathology, personality disorder, or obsessive-compulsive disorder made by an accredited professional.

**Table 1:** Breakdown of students per group/subgroup.

Intelligence	Age groups			
	12–14		15–17	
HIA	HIA 1	Primary education	HIA 2	Primary education
		Secondary education		Secondary education
AIC	Group 1		Group 2	

### Instruments

Almost Perfect Scale Revised (APS-R) by Arana et al. (2006). It consists of 23 items designed to assess adaptive and maladaptive aspects of perfectionism using a Likert-type response format. APS-R comprises three subscales: Standards, Order, and Discrepancy. The factorial structure of the scale has been confirmed through exploratory and confirmatory factor analyses. Cronbach’s alpha coefficients were 0.92 for Discrepancy, 0.85 for Standards, and 0.86 for Order. In this research, the distinction between adaptive and maladaptive perfectionism can be made using the Multidimensional Perfectionism Scale (APS-R), which evaluates three dimensions: High Standards, Order, and Discrepancy. Adaptive perfectionism is characterized by high standards and low levels of discrepancy, while maladaptive perfectionism is associated with high standards combined with high discrepancy or with pronounced concerns about mistakes and doubts regarding one’s actions.

Ryff’s Psychological Well-being Scale (Díaz et al., 2006). This instrument consists of 39 items grouped into 6 dimensions or subscales: self-acceptance, positive relations with others, autonomy, environmental mastery, purpose in life, and personal growth. Specifically, the reduced version developed in the Spanish adaptation has been used. The scales demonstrated good internal consistency, with Cronbach’s alpha (*α*) values ranging from 0.71 to 0.83, except for the personal growth scale, which showed an internal consistency of 0.68.

The Family Functionality Questionnaire (FF-SIL), developed by Ortega et al. (1999), is a widely used tool for assessing family functioning. This instrument consists of 14 items covering seven key dimensions of intrafamilial relationships: cohesion, harmony, communication, affectivity, roles, permeability, and adaptability. Each dimension is assessed through two questions using a five-point Likert scale ranging from “almost never” to “almost always.” The scores obtained allow families to be classified into four categories: functional, moderately functional, dysfunctional, and severely dysfunctional. The FF-SIL demonstrates high internal consistency (Cronbach’s alpha = 0.85), indicating strong item homogeneity despite its multidimensional nature. This variable is relevant as it relates to the stimulation of cognitive abilities and the potential of HIA children.

Socialization Battery BAS-3 by Silva and Martorell (2024). This battery gathers information regarding the various social contexts in which individuals interact. It comprises a total of 75 items aimed at adolescents aged 11 to 19 years, allowing for a profile of social behavior based on five scales, demonstrating satisfactory internal consistency. Regarding score levels, they remain stable over moderate intervals (consideration for others, self-control in social relations, social withdrawal, social anxiety/shyness, leadership, and sincerity). The study by Garaigordobil and Oñederra (2010) on the BAS-3 showed internal consistency values for a sample of Spanish children, achieving a total Cronbach’s alpha reliability of 0.79.

Rosenberg Self-Esteem Scale (González et al., 2000). The Rosenberg self-esteem test, with well-established psychometric properties, is a widely used tool for assessing self-esteem in clinical and scientific settings. It consists of 10 questions with scores ranging from 1 to 4, resulting in a total score from 10 to 40. The questions assess self-worth and the level of self-satisfaction. To control for acquiescence bias, five statements are positively formulated and five are negatively formulated. Its inclusion among the study variables was due to the influence of self-esteem on overall well-being and identity formation during adolescence (Robins et al., 2001). A bifactor analysis of the RSES was conducted in schoolchildren, confirming its bidimensionality, and linking it to physical activity and psychological well-being (Chacón-Borrego et al., 2022). Its Cronbach’s alpha was 0.87.

An *ad hoc* questionnaire was designed using Google Forms to collect data and gather information on whether there was academic demotivation and poor educational performance. It also examined whether parenting styles or upbringing patterns were characterized by high parental expectations. Additionally, sociodemographic information related to economic and cultural levels was collected.

### Procedure

To enable the participation of families of adolescents in the sample, the following materials were sent online: an information and consent form regarding their children’s involvement in the research, a structured interview to be completed, and a questionnaire on family functioning.

The participating students, identified as having high intellectual abilities (HIA) in the province of Cádiz, voluntarily attended extracurricular mentoring sessions. These sessions were organized during the 2020/2021 academic year by the Andalusian Regional Ministry of Education and Sports, in collaboration with the University of Cádiz. Students registered for these sessions through their school counsellors, based on their individual interests.

All participating students had previously been identified as HIA in public, private, or semi-private schools, either by one of the study’s authors or by certified technical staff. During a phone call, families were informed that their children could, independently of their participation in the mentoring sessions, voluntarily take part in a research study on individuals with high intellectual abilities. Participation required both the student’s voluntary assent and the legal guardian’s informed consent.

They voluntarily attended two after-school tutoring sessions, each lasting three and a half hours per day, during which the research data collection instruments were administered. During a telephone call to the families, relatives were informed that their children could voluntarily participate in the research on individuals with high intellectual abilities. This participation was entirely independent of their attendance at the mentoring sessions, although it could take place in the same setting. Participation required both the student’s voluntary assent and the informed consent of their legal guardian.

Similarly, these students were contacted through their schools’ guidance departments to gather additional information about their characteristics. Other adolescents with high intellectual abilities also participated by attending sessions held at the Faculty of Education Sciences (Bay of Cádiz). These students had previously been assessed and identified as HIA through the High Abilities Association (ASUC), sometimes even several years before the research began.

These participants were also contacted by phone and informed that, as a gesture of appreciation for their involvement in the study, they would be offered a voluntary mentorship session on self-awareness, conducted by the researcher. All participants underwent the same type of assessment tests administered by accredited expert personnel from the Department of Psychology or by one of the authors of this study. The tests were conducted under appropriate conditions, following the application manuals’ instructions. There was no issue with students from any group participating in the research attending the mentoring sessions, as these were always scheduled in the afternoon, outside regular school hours. Students in the average ability group—whose intellectual level was confirmed through the study’s administered assessments and who were identified by their tutors as showing no indicators of high ability—could also be selected for participation. These students were informed that their involvement was entirely voluntary and aimed at contributing to a study intended to demystify negative stereotypes associated with high intellectual abilities. The assessments for this group were conducted in the school’s Guidance Department to verify the tutors’ observations that the students did not present signs of disability or mental disorder and to evaluate their intellectual ability. All evaluations were carried out strictly on a voluntary basis, following the informed consent of their legal guardians. Participation was explicitly authorized by the school’s management team. It was clearly explained to all participants that their results would be treated anonymously and would have no influence on educational actions or decisions concerning them. Evaluations were scheduled so as not to interfere with regular academic activities. The evaluation was conducted individually over two or three sessions, each lasting approximately 50 min, to minimize fatigue.

Students considered to be at high social risk did not participate in the study. Typically, secondary school students presenting with severe psychosocial difficulties, psychopathological symptoms, clinical conditions, or mental disorders are referred to social services or mental health professionals. This referral process is managed by school counsellors, who receive regular updates through weekly meetings with tutors. These meetings allow for the continuous identification, monitoring, and coordination with external support services. As such, these students were not included in the current research.

Specifically, students diagnosed with obsessive-compulsive disorder or any other mental disorder were excluded from participation in advance. However, psychological well-being itself was not used as an exclusion criterion. On the contrary, it was a variable of interest in the study, particularly in exploring its relationship with the multidimensional construct of perfectionism—understood here as a personality trait that does not necessarily imply psychopathology in students with high intellectual abilities.

#### Design

This is a descriptive correlational study with measures of dependent variables and a cross-sectional nature allowing analysis of the behavior of study variables across different age groups. As a screening criterion, AIC participants were those who scored within average percentiles (PC < 75). The HIA groups were selected according to the legal framework of the autonomous community of Andalusia, which defines HIA students as those who “manage” and relate multiple cognitive resources of logical, numerical, spatial, memory, verbal, and creative types with a given score, classifying them into the categories of Intellectual Giftedness (high level, above the 75th percentile in all assessed abilities), Complex Talent (combination of several abilities with a percentile above 80 in at least three cognitive capacities), or Simple Talent, referring to high aptitude or competence in a specific area (above the 95th percentile). The identification of HIA subjects was carried out using Renzulli (2016)'s tri-dimensional model, recognized as the standard in the psycho-pedagogical evaluation conducted in our school context, based on the following three variables: intellectual capacity, perseverance, and creativity according to the described tests.

#### Ethical statement

Before administering the instruments used, all participant families were informed about the research conditions, confidentiality rights, and data protection procedures. Written informed consent was obtained from families before minors participated voluntarily, complying with research ethics standards. The data obtained have been processed anonymously to always preserve the privacy of participants.

### Data analysis

The statistical analyses conducted included several methods. Inferential statistics were used to compare means, such as t-tests (e.g., for psychological well-being between groups) and chi-square tests (e.g., for perfectionism distribution). Correlation analysis (Pearson) was applied to explore relationships between socialization factors and psychological well-being. Finally, a Structural Equation Model (SEM) with Bayesian methods examined the relationships between perfectionism types and social functioning. R^2^ values and *p*-values indicated the variance explained and statistical significance.

## Results

To offer a comprehensive overview of our sample, Table 1 displays the descriptive statistics for each variable, categorized by their respective group and subgroup.

### Perfectionism

Statistically significant differences were found between the means of perfectionism according to the intellectual capacities of the subjects, with the mean being higher in the case of the HIA groups (*p* < 0.05; *F* = 3.243; *p* < 0.001; R^2^ = 0.642). Additionally, the HIA subjects exhibited not only adaptative perfectionism, but also maladaptive perfectionism compared to the absence of perfectionism.

**Figure 1:**
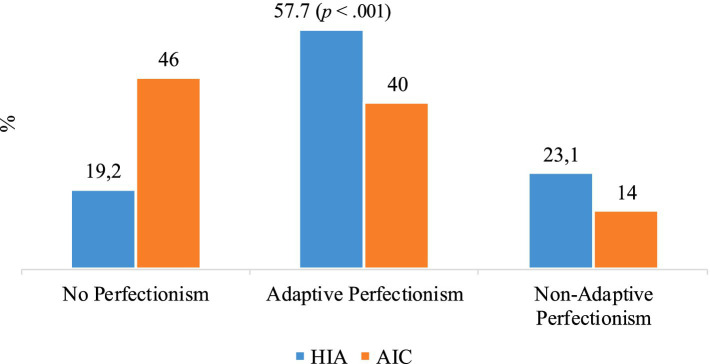
Type of perfectionism by intellectual capacity (HIA/AIC).

For the HIA participant group, 19.2% of participants did not exhibit any form of perfectionism. Adaptive perfectionism was found in 57.7% of the participants, while maladaptive perfectionism was present in 23.1%. In contrast, among the AIC subjects, 46.0% did not exhibit perfectionism, adaptive perfectionism was present in 40.0% of the cases, and maladaptive perfectionism in the remaining 14.0%. These differences were statistically significant (*χ*^2^ = 8.40; *p* < 0.01). The distribution of perfectionism types varies according to intellectual level. Both adaptive and maladaptive perfectionism proportions were higher in the HIA group compared to the average. There was a wide percentage of responses, with scores ranging from a minimum of 34 to a maximum of 144 (see Figure 1).

### Psychological well-being

The average psychological well-being score was 142.45 points across the sample, which, according to the Ryff scale, is at the beginning of the range considered high well-being. Overall, 55.3% had a high degree of psychological well-being, and only 6.8% had low well-being. When comparing the mean values of psychological well-being, a higher average was observed in the HIA subjects (145.04 vs. 139.80), although the differences between the groups were not statistically significant (*t* = 1.8; *p* ns) (Table 2).

**Table 2:** Means, standard deviations, minimums, and maximums of key psychological and social variables according to intellectual capacity and perfectionism type.

Variable	Mean	SD	Min	Max
Psychological Well-being (HIA)	145.04	14.68	122.00	168.00
Psychological Well-being (AIC)	139.80	15.01	122.00	168.00
Self-Esteem (HIA)	31.37	4.94	10.00	40.00
Self-Esteem (AIC)	30.70	4.61	10.00	40.00
Social Self-Control (HIA)	10.48	2.78	0.00	15.00
Social Self-Control (AIC)	10.04	3.43	0.00	15.00
Sincerity (HIA)	6.21	1.95	0.00	10.00
Sincerity (AIC)	5.38	2.04	0.00	10.00
Social Anxiety/Shyness - No-P (HIA)	3.40	3.44	0.00	12.00
Social Anxiety/Shyness - AP (HIA)	4.60	3.06	0.00	12.00
Social Anxiety/Shyness - NAP (HIA)	5.50	3.12	0.00	12.00
Social Retraction - No-P (HIA)	1.40	1.58	0.00	12.00
Social Retraction - AP (HIA)	3.50	3.35	0.00	12.00
Social Retraction - NAP (HIA)	5.75	3.86	0.00	12.00
Family Functioning - Functional (HIA)	63.50	33	0.00	100.00
Family Functioning - Functional (AIC)	37.30	19	0.00	100.00

HIA = High Intellectual Ability; AIC = Average Intellectual Capacity; No-P = No Perfectionism; AP = Adaptive Perfectionism; NAP = Non-Adaptive (Maladaptive) Perfectionism. Min and Max refer to the minimum and maximum scores observed or possible for each measure.

Regarding the APS-R scale that assesses perfectionism and its different types, no statistically significant differences were found between the groups of participants (*F* = 1.31, *p*, ns), suggesting that the means of psychological well-being do not differ between the three types of perfectionism: (*No-perfectionism, M* = 140; *SD* = 13.01; *Adaptive Perfectionism M* = 147.77; *SD* = 14.35; *Non-Adaptive Perfectionism M* = 142.42, *SD* = 16.33). In relation to age and psychological well-being expressed by the participants, the differences between the different age groups based on intellectual capacity were not significant (*F* = 1.26; *p* = *ns*; *R*^2^ = 0.08).

### Self-esteem

The average self-esteem score in the total sample was nearly 31 points, indicating that according to the Rosenberg scale norms, the study group had good self-esteem. The mean self-esteem score was 31.37 for the HIA group and 30.70 for the AIC group. No statistically significant differences were found indicating that the means of both groups differ based on the two types of intelligence studied (*Z* = 0.230; *p* ns; *R*^2^ = 0.005). However, when comparing self-esteem levels between the two groups, more cases of low self-esteem were observed among HIA subjects (21.2% vs. 9.8%) as well as high self-esteem (11.5% vs. 10%). Meanwhile, participants in the AIC group exhibited more cases of moderate to good or normal self-esteem (68.6% vs. 48.1%).

### Socialization

In the numerical expression of the variables from the BAS-3 Socialization Battery, it can be observed that there was no statistical significance, nor notable effect size (almost negligible: < 1%), indicating differences between the AIC and HIA groups. With one exception in the scores of the sincerity scale, where statistically significant differences were observed (*p* < 0.05), the mean value for the HIA group was higher compared to the mean of the AIC subjects (6.21 vs. 5.38; with a moderate-to-low effect: 4.2%) (Table 3).

**Table 3:** Inferential analysis.

BAS-3 socialization	HIA	AIC			
	Mean (SD)	Mean (SD)	*t*	*p*	*R* ^2^
Consideration of others	11.85 (2.19)	11.92 (1.47)	0.04	0.843	0.000
Social self-control	10.48 (2.78)	10.04 (3.43)	0.51 ^NS^	0.477	0.005
Social retraction	3.62 (±3.48)	4.00 (3.43)	0.32 ^NS^	0.575	0.003
Social anxiety/shyness	4.58 (±3.16)	4.78 (3.22)	0.10 ^NS^	0.749	0.001
Leadership	6.73 (±3.30)	6.92 (2.75)	0.10 ^NS^	0.754	0.001
Sincerity	6.21 (1.95)	5.38 (2.04)	4.42*	0.038	0.042

Contrast of the variables from the BAS-3 socialization battery, based on intellectual capacity. (*) *p* < 0.001.

Table 4 presents Pearson correlation coefficients between socialization factors and psychological well-being, comparing two groups with different intellectual capacities: HIA (*n* = 52) and AIC (*n* = 50). It is observed that “Social Self-Control” shows a positive correlation in the high-ability group (0.31) and a negative correlation in the average-ability group (−0.14), with a statistically significant difference (*p* < 0.023). This suggests that, in individuals with high intellectual abilities, better social self-control is associated with higher psychological well-being, whereas in those with average abilities, this may not be the case. Other factors such as “Consideration of Others,” “Social Retraction,” and “Social Anxiety/ Shyness” did not show significant differences between the groups.

**Table 4:** Associative analysis.

Psychological well-being (BAS-3)	Correlation			
	HIA	AIC	*F*	*p*
Consideration of others	0.21	0.11	0.21	0.613
Social self-control	0.31	−0.14	2.27	0.023*
Social retraction	0.12	−0.08	0.99	0.323
Social anxiety/shyness	0.07	−0.05	0.59	0.554
Leadership	0.20	0.24	0.21	0.836
Sincerity	−0.07	−0.02	0.25	0.805

Pearson correlation coefficients: socialization and psychological well-being. comparison between intellectual capacity groups (HIA/AIC). (*) *p* < 0.001.

Regarding the type of perfectionism and socialization, Table 5 shows an inferential analysis of the BAS-3 scales based on the type of perfectionism in students with high intellectual abilities. There were no significant differences found in “Social Anxiety/Shyness” between types of perfectionism (*Z* = 2.304, *p* = 0.105, *R^2^* = 0.044), although anxiety is higher in non-adaptive perfectionism (5.50) compared to adaptive (4.60) and no perfectionism (3.40). Similarly, “Social Retraction” is higher in non-adaptive perfectionism (5.75), followed by adaptive (3.50) and no perfectionism (1.40), but without significant differences (*Z* = 2.147, *p* = 0.122, *R^2^* = 0.0412). Scores on “Consideration for Others” and “Leadership” show no significant differences between groups. Overall, non-adaptive perfectionism is associated with greater social difficulties, although the results were not statistically significant.

**Table 5:** Inferential analysis.

BAS-3	Perfectionism type	Mean (SD)	*t*	*p*	*R*^2^
Total score social anxiety/shyness	No-P	3.40 (3.44)	2.304	0.105	0.044
	AP	4.60 (3.06)			
	NAP	5.50 (3.12)			
Total score social retraction	No-P	1.40 (1.58)	2.147	0.122	0.0412
	AP	3.50 (3.35)			
	NAP	5.75 (3.86)			
Total score consideration of others	No-P	12.10 (1.29)	1.098	0.338	0.022
	AP	11.67 (2.71)			
	NAP	12.08 (1.16)			
Total score leadership	No-P	6.60 (3.72)	1.118	0.331	0.022
	AP	7.03 (2.91)			
	NAP	6.08 (4.01)			

BAS-3 scales based on type of perfectionism in high intellectual ability students. No-P, no perfectionism; AP, adaptative perfectionism; NAP, non-adaptative perfectionism.

### Family functioning

Considering all the different categories of family functionality assessed with the FF-SIL questionnaire, the differences between the HIA and AIC groups were significant (*F* = 14.09; *p* < 0.005). No factors related to the composition and functioning of families were found to be affecting the results obtained in the research (Table 6), as family functionality predominated in both study groups and even the family climates available to HIA participants were advantageous. This finding is consistent with the overall level of psychological well-being exhibited by the study participants.

**Table 6:** Inferential analysis.

Family functioning	HIA % (Frequency)	AIC % (Frequency)	*X^2^*	*p*	*R* ^2^
Funtional	63.5 (33)	37.3 (19)	14.09	0.003	0.137
Moderately functional	25.0 (13)	51.0 (26)			
Moderately dysfunctional	7.7 (4)	0 (0)			
Dysfunctional	3.8 (2)	11.8 (6)			

Contrast of family functioning categories based on high intellectual abilities (HIA) or average intellectual capacity (AIC) groups.

#### Structural equation model (SEM)

**Figure 2:**
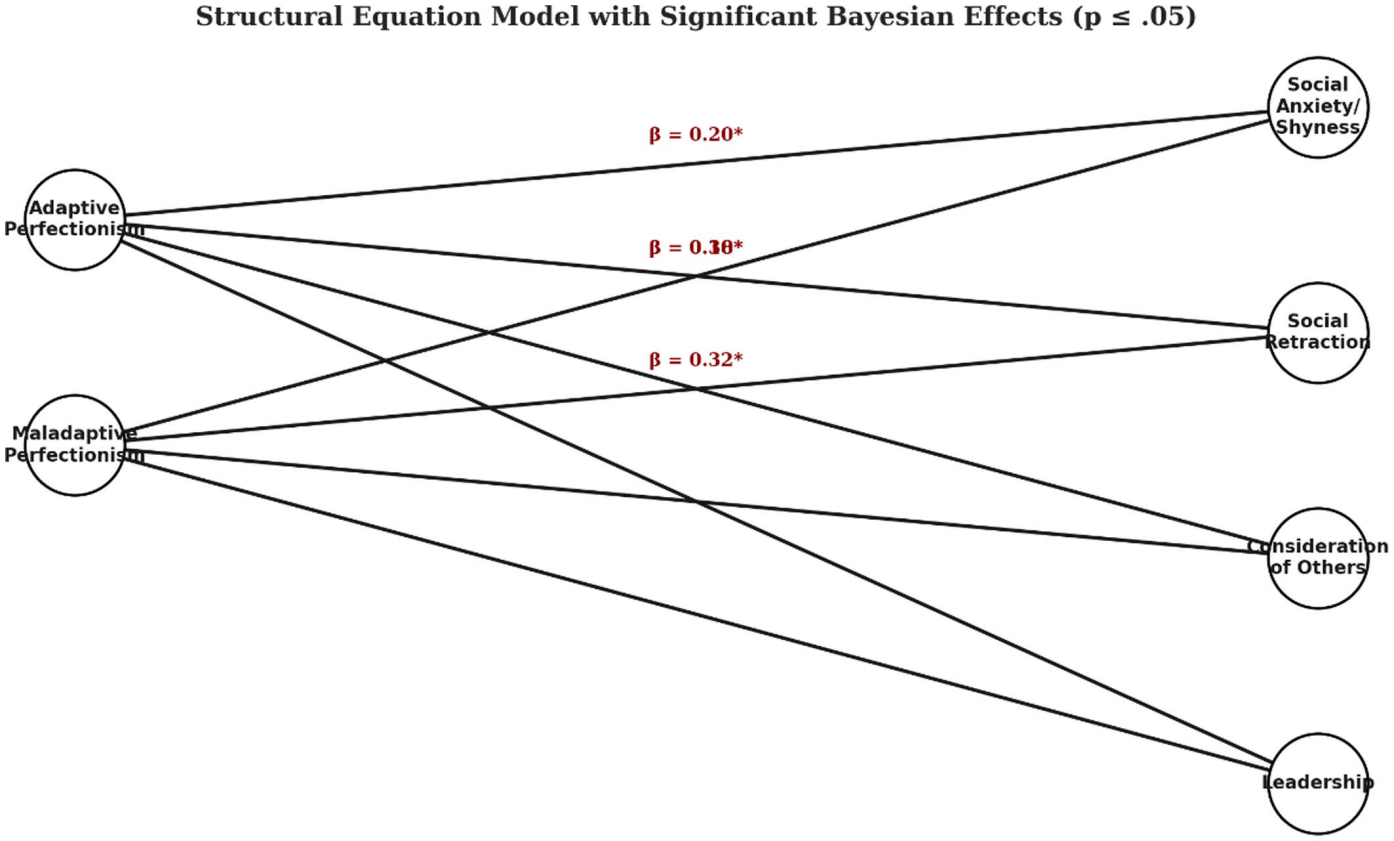
Structural equation model (SEM) diagram reflecting the Bayesian analysis results. Predictor variables: adaptive perfectionism (AP); maladaptive perfectionism (NAP) outcome dimensions: social anxiety/shyness; social retraction; consideration of others; leadership.

A Structural Equation Model (SEM) within a Bayesian framework was implemented to jointly examine the relationships between perfectionism type—coded into three groups (No-Perfectionism [No-P], Adaptive Perfectionism [AP], and Maladaptive Perfectionism [NAP])—and the various dimensions of social functioning assessed via the BAS-3 (Social Anxiety/Shyness, Social Retraction, Consideration of Others, and Leadership). The procedure allowed for the development of the following elements to configure the Bayesian model (see Figure 2).

##### Model specification

Observed variables: scores for each BAS-3 dimension were modeled as observed variables; Grouping variable: perfectionism type was converted into dummy variables, with the No-P group serving as the reference; Regression model: direct paths were specified from the dummy variables to each BAS-3 indicator to evaluate the specific effects of AP and NAP relative to No-P.

##### Bayesian approach

Non-informative priors (e.g., normal distributions with a mean of 0 and large variances) were defined for the model parameters, allowing the data to update the estimates objectively; Inference was conducted using Markov Chain Monte Carlo (MCMC) chains, with parameter convergence verified via the R-hat index (expected value < 1.1).

##### Model evaluation

The estimated parameters (slopes and residual variances) were analyzed, and 95% credibility intervals were computed for each effect; Model fit was assessed using Bayesian criteria (such as the Deviance Information Criterion [DIC]), which facilitated the comparison of alternative models, including those incorporating potential mediating relationships or additional covariates.

##### Integration of previous results

Preliminary univariate analyses (using t-values, *p*-values, and *R*^2^) indicated differences in the expected direction—for example, higher scores in social anxiety/shyness and social retraction for the NAP group—but these effects showed marginal significance and limited explained variance (*R*^2^ between 0.022 and 0.044). The multivariate Bayesian model allowed integration of these dimensions and examination of their covariance, yielding a more comprehensive and robust picture of perfectionism’s influence.

Table 7 below summarizes the results of the SEM analyzed. In this model, the direct relationships between perfectionism type—coded into two contrasts (Adaptive Perfectionism [AP] and Maladaptive Perfectionism [NAP], with No-Perfectionism [No-P] as the reference group)—and the various dimensions evaluated by the BAS-3 were examined. Parameters are expressed as standardized estimates, accompanied by their 95% credibility intervals and p-values indicative of the evidence for each effect. R^2^ values represent the proportion of variance explained in each dimension.

**Table 7:** Results of the structural equation model (SEM) – Bayesian approach.

Dimension	Comparison	Standardized estimate (β)	95% Credibility interval	*p*	*R* ^2^
Social anxiety/shyness	AP vs. No-P	0.20	[0.02, 0.38]	0.05	0.044
	NAP vs. No-P	0.30	[0.12, 0.48]	0.02	
Social retraction	AP vs. No-P	0.18	[0.01, 0.35]	0.04	0.041
	NAP vs. No-P	0.32	[0.14, 0.50]	0.01	
Consideration of others	AP vs. No-P	−0.10	[−0.27, 0.07]	0.25	0.022
	NAP vs. No-P	0.02	[−0.15, 0.19]	0.80	
Leadership	AP vs. No-P	0.12	[−0.05, 0.29]	0.15	0.022
	NAP vs. No-P	−0.08	[−0.25, 0.09]	0.30	

[AP] adaptive perfectionism; [NAP] maladaptive perfectionism; [No-P] No-perfectionism.

## Discussion and conclusion

The research on perfectionism has evolved from a negative view to a multidimensional adaptive perspective. Although historically associated with psychological problems, today its adaptive potential is recognized, especially in individuals with high intellectual abilities (HIA). This population, often stigmatized, demonstrates metacognitive skills that allow them to manage perfectionism in a healthy manner, linked to greater success and well-being. However, the relationship between perfectionism and HIA is still debated, along with the impact of cultural and educational context on the development of both. This study explored the relationship between high intellectual ability (HIA) and perfectionism, distinguishing between adaptive and maladaptive forms. It examined how perfectionism influences psychological well-being in HIA adolescents and the moderating effects of contextual factors.

The findings on the characteristics of perfectionism in adolescents with HIA in this study can contribute to answering a question that was previously unresolved and required additional evidence according to various authors (Yi and Gentry, 2021). Thus, the most significant findings were found when analyzing the frequency of perfectionism types in the two groups (HIA and AIC) using the APS-R. In this way, differences have been found compared to previous studies. This includes perspectives maintaining a negative view of perfectionism, as well as more recent ones concluding that 70% of minors with HIA exhibit adaptive perfectionism or a tendency towards it, contrasting with non-adaptive perfectionism in individuals with HIA (Stricker et al., 2019). Recent studies propose a dual aspect of the perfectionism construct, highlighting its association with characteristics linked to psychological well-being in many instances (Duplenne et al., 2024).

Sastre (2019) in multivariate analyses, already obtained results indicating that students with HIA tend to be more perfectionistic than average-intelligence students, without significant differences regarding whether their perfectionism was healthier or unhealthier. In our study, in addition to providing evidence of higher frequency of perfectionism in adolescents with HIA compared to AIC, as a novelty, it also suggests that the former group exhibits high levels at both ends of the perfectionism spectrum (adaptive vs. non-adaptive), which could explain why psychological well-being outcomes are nearly comparable to those of the AIC group. Participants with HIA showed a slightly higher average than those with AIC. However, the results are inconclusive as they do not reach statistical significance. The same applies to the analysis of mean psychological well-being across different age ranges, where differences favouring HIA were observed but are not significant.

These results may suggest that individuals with HIA not only exhibit personal well-being comparable to that of AIC individuals but also a tendency towards higher well-being, as suggested by Hornstra et al. (2023). On the other hand, the lack of statistically significant differences in psychological well-being measures between groups based on types of perfectionism supports the notion that individuals with HIA are not necessarily linked to maladaptive personality traits (Neihart and Yeo, 2018). It also does not indicate a higher risk of psychological disorders or issues related to psychological well-being compared to AIC students (Duplenne et al., 2024).

Regarding self-esteem, again, no differences were found in the average values between the two groups. However, when comparing the levels of self-esteem assessed with the RSES, differences were observed. There were more cases in the HIA groups, both with high self-esteem, in line with studies by Wang et al. (2012), and with low self-esteem. These results align with those related to perfectionism, which is precisely marked by the tendency to judge one’s self-esteem based on the ability to achieve set goals (Fındık and Afat, 2023). Self-esteem in individuals with HIA, due to its multidimensional nature, is influenced by both external and internal factors. In this sense, the suggestion would be that strengthening this dimension is important, particularly in individuals with HIA.

In the parameters evaluated by the BAS-3 socialization battery concerning intellectual capacity, no statistically significant differences were found in the total instrument scores. However, a positive correlation was found between social self-control and psychological well-being for adolescents with HIA. In the AIC group, this correlation was not significant and was negative. Similarly, a positive correlation was observed in the HIA groups regarding consideration for others and psychological well-being. Lastly, in terms of leadership intensity, the correlation was similar and favourable for both the HIA and AIC groups regarding psychological well-being. Although no significant differences were found in the overall assessment of socialization between HIA and AIC, notable correlations were found in specific subcategories. Adolescents with HIA showed a positive correlation between social self-control and psychological well-being, whereas in AIC, this correlation was negative. Additionally, adolescents with HIA obtained positive correlations between consideration for others and psychological well-being. Regarding leadership intensity, both the HIA and AIC groups showed favourable correlations with psychological well-being. Despite the lack of overall differences, adolescents with HIA displayed specific correlations between social self-control, social consideration, and psychological well-being.

The Structural Equation Model (SEM) using a Bayesian approach provides a nuanced understanding of the relationship between intellectual capacity, perfectionism types, and psychological well-being, addressing the study’s three key objectives. According to relationship between Intellectual Capacity and Perfectionism (objective 1), the model highlights how perfectionism manifests differently among adolescents with high intellectual ability (HIA) compared to those with average intellectual capacity (AIC). The inclusion of adaptive perfectionism (AP) and maladaptive perfectionism (NAP) as separate constructs allows for an examination of their distinct effects. The significant positive associations between NAP and social anxiety/shyness (*β* = 0.30, *p* < 0.02) and social retraction (*β* = 0.32, *p* < 0.01) suggest that HIA adolescents who exhibit maladaptive perfectionism may experience heightened emotional distress, aligning with previous findings on the psychological vulnerabilities of perfectionistic gifted students (Sand et al., 2021).

The SEM results indicate that perfectionism influences different aspects of psychological well-being (objective 2). While AP showed only minor associations with social anxiety (*β* = 0.20, *p* < 0.05) and social retraction (*β* = 0.18, *p* < 0.04), NAP was more strongly related to negative psychological outcomes. The lack of significant relationships between perfectionism and leadership or consideration of others suggests that perfectionism primarily affects emotional and social distress rather than prosocial behaviors.

Finally, considering the contextual moderators of Perfectionism’s Effects (objective 3), although the SEM primarily focuses on direct effects, the relatively low explained variance (*R*^2^ between 0.022 and 0.044) suggests the presence of unmodeled moderating variables, such as cultural or educational factors. These findings reinforce the need for further exploration of contextual influences, as prior research suggests that cultural attitudes toward achievement and social expectations may shape how perfectionism develops in HIA adolescents. Overall, the Bayesian SEM provided empirical support, reinforcing the complex interplay between perfectionism, intellectual ability, and well-being. While adaptive perfectionism may contribute to the development of social anxiety and retraction to some extent, other contextual, personal, or environmental factors not included in the model may also play a relevant role. We consider these findings meaningful, as they reflect the multi-factorial complexity of social behaviors in students and underscore the need for future studies to incorporate additional variables (e.g., emotional regulation, social support) to improve the explanatory power of the models and clarify the protective role of adaptive perfectionism in educational contexts.

Some limitations of this study are related to the sample and the screening process used for its selection. The group of HIA participants was initially identified by teachers. Although educational authorities administer screening questionnaires to refine the process and assist teachers in the initial phases of detecting HIA, not all referrals from the actual population with HIA may reach the specialists who conduct psychopedagogical evaluations. Therefore, we consider that there is a limitation in the selection of the population that truly represents the study’s target. In other words, this screening system may potentially exclude students with HIA, thereby limiting the sample size.

This research opens up a promising field of study and raises questions that need further analysis. It is necessary to deepen our understanding of the psychoeducational variables that contribute to the development of adaptive perfectionism, which is of interest to the scientific community due to the scarcity of existing studies, particularly those referring to gifted and talented individuals (Gonzálvez et al., 2016). This also has practical implications in the educational process of gifted students.

Attributing any failures of gifted individuals solely to their own characteristics ceases to make sense when considering the influence of the educational context on their development. In the school setting, it is evident that gifted students typically do not face academic problems if their needs are met (Hornstra et al., 2023). However, gifted students often experience educational models during their schooling that are not well-suited to their educational needs, facing undifferentiated curricula marked by repetitive and slow-paced learning (Algaba and Fernández, 2021). This practice can lead to apathy, boredom, and even aversion among gifted students (Martínez and Guirado, 2010).

The educational response for these students should be more personalized; the curriculum should be tailored to their profile, personality, and socio-educational context to ensure their inclusion and full potential development (Sternberg, 2024). Although giftedness tends towards excellence, it can be affected in its expression by maladaptive perfectionism, which can lead to dysfunction in managing intellectual potential (Sastre, 2024). Given that gifted individuals expressing adaptive perfectionism often achieve success with little experience of failure, future research on identifying these profiles early on is crucial to support talent development.

On the other hand, considering our results indicating higher levels of maladaptive perfectionism coexisting in the gifted population, there is a pressing need for future studies on the role of perfectionism not only in academic underachievement but also in early school dropout, low academic performance, and even school refusal (Smith et al., 2022). All of these insights would contribute to designing intervention programs for gifted youth, providing them with tools to manage adaptive perfectionism profiles, emphasizing personal effort, collaborative learning, and creativity, thereby avoiding the inhibition of their potential.

Some educational implications of this study highlight the need for tailored interventions for HIA adolescents with perfectionism. Early detection of maladaptive perfectionism is crucial due to its impact on anxiety and social withdrawal, a concern emphasized in previous research (Balestrini and Stoeger, 2024; Raoof et al., 2024). Personalized socio-emotional support, including mindfulness and cognitive-behavioral therapy, is recommended. Additionally, a flexible academic environment that prioritizes learning over perfection can help mitigate perfectionism’s negative effects. Finally, interventions should account for contextual and cultural factors to ensure their effectiveness.

## Educational and social implications

Social anxiety and social withdrawal—often manifested through excessive concern over making mistakes, fear of failure, and a heightened need for external validation—can lead to avoidance of social situations due to fear of judgment or rejection. In this study, such symptoms are observed among participants identified with the maladaptive perfectionism typology. These findings are consistent with previous research, particularly among students with high intellectual abilities (HIA), as noted by authors such as Balestrini and Stoeger (2024) and Raoof et al. (2024).

In contrast, among high-ability students who exhibit adaptive perfectionism, the data reveal only weakly significant associations with social anxiety and withdrawal. This may be due to the complex interplay between high intellectual ability and the internal and external expectations that often accompany it. Even adaptive perfectionism—typically associated with positive traits—may fail to offer full protection, as the persistent pressure to excel can hinder the development of self-appreciation and self-compassion.

It is therefore essential to actively foster self-compassion in high-ability students, helping them to strike a healthy balance between the pursuit of excellence and the maintenance of their mental well-being (Turanzas et al., 2020). Self-compassion is a key skill that can play a crucial role in the development of preventive and educational intervention programmes for students, regardless of the type of perfectionism they exhibit—whether adaptive or maladaptive. It is essential to promote family, school, and social practices that foster self-compassion or related competencies, particularly for individuals with high intellectual abilities. These practices can support them in managing pressure, perfectionist tendencies, and associated stress, ultimately enabling them to better realise their potential. By cultivating self-compassion, students are more likely to develop emotional resilience, enhance their self-esteem, and respond to challenges with greater effectiveness and psychological well-being.

Given the more severe implications of maladaptive perfectionism, early detection is essential for adolescents with high intellectual abilities (HIA), due to the significant impact it can have on their well-being. In such cases, it is particularly important to provide personalised socio-emotional support, including strategies such as mindfulness and cognitive-behavioural therapy. Moreover, fostering a flexible academic environment that emphasises learning and growth over perfection can help reduce the negative consequences associated with perfectionism. Finally, any intervention should be sensitive to contextual and cultural factors to ensure its relevance and effectiveness (Fernández-Molina and Rivera-Gallardo, 2025). It is crucial to understand the underlying dynamics of perfectionism in high-ability students and to offer individualised support that helps them develop a healthy relationship with it. In summary, perfectionism in students with high intellectual abilities can be a double-edged sword. Educators play a key role in guiding these students by fostering self-compassion, experiential learning, and emotional resilience. These approaches enable students to reach their full potential without being negatively impacted by internal pressures or expectations from their school and family environments (Medina-Castro et al., 2024).

A supportive family environment is equally essential. Emotional warmth, open communication, and acceptance of the unique characteristics associated with high ability are fundamental to the psychological well-being of these students. Parenting styles that promote autonomy, independence, and the exploration of personal interests—while also providing appropriately challenging opportunities—can contribute significantly to positive development. Additionally, families that communicate effectively and manage conflict constructively can enhance a child’s emotional regulation and capacity to form healthy relationships.

Family functioning that maintains realistic expectations regarding academic performance and social development—without placing undue pressure on the child—can be particularly beneficial. Moreover, the family can serve as a vital source of social support, encouraging the development of external support networks and social skills (Zamudio et al., 2024).

The challenges faced by highly able students stem not from their innate abilities, but from how the education system and society respond to their specific needs. In many cases, their potential remains unrealised due to the inability of educational and social environments to adequately adapt to their unique learning and developmental requirements.

## Data Availability

The raw data supporting the conclusions of this article will be made available by the authors, without undue reservation.

## References

[ref1] Algaba A. Fernández T. (2021). Características socioemocionales en población infanto-juvenil con altas capacidades: una revisión sistemática. Revista de Psicología y Educación. 16, 60–74. doi: 10.23923/rpye2021.01.202

[ref2] Arana F. Scappatura M. L. Lago A. Keegan E. (2006). Adaptive and maladaptive perfectionism and psychological distress in Argentine university students: An exploratory study using the APS-R. Paper presented at the XIV Research Conference of the Mercosur, Faculty of Psychology, University of Buenos Aires, Buenos Aires, Argentina.

[ref3] Balestrini D. P. Stoeger H. (2024). Cultural framing of giftedness in recent US fictional texts. PLoS One 19:e0307222. doi: 10.1371/journal.pone.0307222, PMID: 39208213 PMC11361604

[ref4] Baudson T. G. Preckel F. (2016). Teachers’ conceptions of gifted and average-ability students on achievement-relevant dimensions. Gifted Child Quarterly 60, 212–225. doi: 10.1177/0016986216647115

[ref5] Beljan P. Gardner J. M. Homaijani D. (2024). Processing speed in gifted children: a clinical neuropsychological perspective. Roeper Rev. 46, 131–139. doi: 10.1080/02783193.2024.2309396

[ref6] Chacón-Borrego F. Gomis-Gomis M. Silva-Sousa C. (2022). Bifactorial analysis of the Rosenberg self-esteem scale and relationship between physical activity and self-esteem in adolescents. Sportis 8, 423–441. doi: 10.17979/sportis.2022.8.3.9152

[ref7] Chan D. W. (2008). Perfectionism and goal orientations among Chinese gifted students in Hong Kong. Roeper Rev. 31, 9–17. doi: 10.1080/02783190802527331

[ref8] Damian L. E. Stoeber J. Negru O. Băban A. (2013). On the development of perfectionism in adolescence: perceived parental expectations predict longitudinal increases in socially prescribed perfectionism. Personal. Individ. Differ. 55, 688–693. doi: 10.1016/j.paid.2013.05.021

[ref9] Díaz D. Rodríguez-Carvajal R. Blanco A. Moreno-Jiménez B. Gallardo I. Valle C.. (2006). Adaptación española de las escalas de bienestar psicológico de Ryff. Psicothema 18, 572–577, PMID: 17296089

[ref10] Duplenne L. Bourdin B. Fernandez D. N. Blondelle G. Aubry A. (2024). Anxiety and depression in gifted individuals: a systematic and meta-analytic review. Gifted Child Quarterly 68, 65–83. doi: 10.1177/00169862231208922

[ref11] Endleman S. Brittain H. Vaillancourt T. (2022). The longitudinal associations between perfectionism and academic achievement across adolescence. Int. J. Behav. Dev. 46, 91–100. doi: 10.1177/01650254211037400

[ref12] Fernández-Mera A. Hinojosa J. A. Duñabeitia J. A. (2024). Exploratory analysis of the differences in emotional intelligence of gifted minors. Electron. J. Res. Educ. Psychol. 22, 11–38. doi: 10.25115/ejrep.v22i62.9226

[ref13] Fernández-Molina M. Rivera-Gallardo N. (2025). Retos Familiares por Alta Capacidad Intelectual: programa de Orientación Familiar. ANDULI. Revista Andaluza de Ciencias Sociales 27, 85–106. doi: 10.12795/anduli.2025.i27.04

[ref14] Fındık H. Afat N. (2023). Perfectionism and life satisfaction in gifted students. Int. J. Psychol. Educ. Stud. 10, 1012–1023. doi: 10.52380/ijpes.2023.10.4.1285

[ref15] Fletcher K. L. Speirs K. L. Finch W. H. Cross T. (2023). Profiles of temperament and perfectionism in high ability college students. Explor. Psychol. Giftedness 2, 11–24. doi: 10.25774/zrps-tb87

[ref16] Fong R. W. Yuen M. (2014). Perfectionism and Chinese gifted learners. Roeper Rev. 36, 81–91. doi: 10.1080/02783193.2014.884202

[ref17] Garaigordobil M. Oñederra J. A. (2010). Inteligencia emocional en las víctimas de acoso escolar y en los agresores. Euro. J. Educ. Psychol 3, 243–256. doi: 10.30552/ejep.v3i2.55

[ref18] González F. A. Sigüenza Y. M. Solá I. B. (2000). Análisis de la dimensionalidad de la Escala de Autoestima de Rosenberg en una muestra de adolescentes valencianos. Revista de Psicología. Universitas Tarraconensis 22, 29–42.

[ref19] Gonzálvez C. Ingles C. J. Vicent M. Sanmartín R. García-Fernández J. M. (2016). Perfeccionismo socialmente prescrito como predictor del alto rechazo a la escuela. J. Develop. Educ. Psychol. 1, 25–32. doi: 10.17060/ijodaep.2016.n1.v1.185

[ref20] Grugan M. C. Hill A. P. Madigan D. J. Donachie T. C. Olsson L. F. Etherson M. E. (2021). Perfectionism in academically gifted students: a systematic review. Educ. Psychol. Rev. 33, 1631–1673. doi: 10.1007/s10648-021-09597-7

[ref21] Hornstra L. Mathijssen A. S. Denissen J. J. Bakx A. (2023). Academic motivation of intellectually gifted students and their classmates in regular primary school classes: a multidimensional, longitudinal, person-and variable-centered approach. Learn. Individ. Differ. 107:102345. doi: 10.1016/j.lindif.2023.102345

[ref22] Kuznetsova E. Liashenko A. Zhozhikashvili N. Arsalidou M. (2024). Giftedness identification and cognitive, physiological and psychological characteristics of gifted children: a systematic review. Front. Psychol. 15:1411981. doi: 10.3389/fpsyg.2024.1411981, PMID: 39635703 PMC11615676

[ref23] Martínez M. Guirado A. (2010). Alumnado con altas capacidades. Barcelona: Graó.

[ref24] Medina-Castro M. Abín A. Fernández E. (2024). Concepción de las Familias y la Escuela Sobre las Altas Capacidades: una Revisión Sistemática. J. Psychol. Educ. 19, 1–10. doi: 10.23923/rpye2024.01.244

[ref25] Mofield E. L. Parker M. (2015). Multidimensional perfectionism within gifted suburban adolescents: an exploration of typology and comparison of samples. Roeper Rev. 37, 97–109. doi: 10.1080/02783193.2015.1008663

[ref9001] Neihart M. (Ed.). (2021). The social and emotional development of gifted children: What do we know? Routledge. doi: 10.4324/9781003238928

[ref26] Neihart M. Yeo L. S. (2018). “Psychological issues unique to the gifted student” in APA handbook of giftedness and talent (pp. 497–510). eds. Pfeiffer S. I. Shaunessy-Dedrick E. Foley-Nicpon M. (Washington DC: American Psychological Association).

[ref27] Noor B. (2023). Pressure and perfectionism: a phenomenological study on parents' perceptions of gifted students' experiences. Front. Educ. 8:1225623. doi: 10.3389/feduc.2023.1225623

[ref28] Ogurlu U. (2020). Are gifted students perfectionistic? A meta-analysis. J. Educ. Gifted. 43, 227–251. doi: 10.1177/0162353220933006

[ref29] Ortega T. de la Cuesta D. Días C. (1999). Propuesta de un instrumento para la aplicación de proceso de atención de enfermería en familias disfuncionales. Rev. Cubana Enferm. 15, 164–168.

[ref30] Pákozdy C. Askew J. Dyer J. Gately P. Martin L. Mavor K. I.. (2023). The imposter phenomenon and its relationship with self-efficacy, perfectionism and happiness in university students. Curr. Psychol. 43, 5153–5162. doi: 10.1007/s12144-023-04672-4, PMID: 40642600

[ref31] Plucker J. A. Callahan C. M. (2014). Research on giftedness and gifted education: status of the field and considerations for the future. Except. Child. 80, 390–406. doi: 10.1177/0014402914527244

[ref32] Raoof K. Shokri O. Fathabadi J. Panaghi L. (2024). Unpacking the underachievement of gifted students: a systematic review of internal and external factors. Heliyon. 10:e36908. doi: 10.1016/j.heliyon.2024.e36908, PMID: 39286082 PMC11402643

[ref33] Renzulli J. (2016). “The three-ring conception of giftedness: a developmental model for promoting creative productivity” in Reflections on gifted education: Critical works by Joseph S. Renzulli and colleagues. ed. Reis S. M. (London: Prufrock Press Inc.), 55–90.

[ref34] Rinn A. N. (2024). A critique on the current state of research on the social and emotional experiences of gifted individuals and a framework for moving the field forward. Gifted Child Quarterly 68, 34–48. doi: 10.1177/00169862231197780

[ref35] Robins R. W. Hendin H. M. Trzesniewski K. H. (2001). Measuring global self-esteem: construct validation of a single-item measure and the Rosenberg self-esteem scale. Personal. Soc. Psychol. Bull. 27, 151–161. doi: 10.1177/0146167201272002

[ref36] Rodríguez R. (2025). El camino a la excelencia: más allá de las altas capacidades intelectuales. ANDULI. Revista Andaluza de Ciencias Sociales 27, 107–131. doi: 10.12795/anduli.2025.i27.05

[ref37] Sand L. Bøe T. Shafran R. Stormark K. M. Hysing M. (2021). Perfectionism in adolescence: associations with gender, age, and socioeconomic status in a Norwegian sample. Front. Public Health 9:688811. doi: 10.3389/fpubh.2021.688811, PMID: 34513782 PMC8424040

[ref38] Saß S. Köller O. Zimmermann F. (2025). Smart but maladapted? Differences in the psychological functioning of intellectually gifted students compared with average-ability students. Gifted Child Quarterly 69, 219–236. doi: 10.1177/00169862241310871

[ref39] Sastre S. (2019). Alta capacidad intelectual: perfeccionismo y regulación metacognitiva. Rev. Neurol. 54, 1–9. doi: 10.33588/rn.54S01.2012011 22374769

[ref40] Sastre S. (2024). Funcionamiento cognitivo y metacognición en la Alta Capacidad Intelectual. Medicina 84, 72–78. 38350628

[ref41] Sastre S. Fonseca E. Ortuño-Sierra J. (2019). From high intellectual ability to genius: profiles of perfectionism. Comunicar 27, 9–17. doi: 10.3916/C60-2019-01

[ref42] Senol F. B. Koca S. Erbasan Ö. (2023). Gifted children’s social problem solving skills, social competence and school adjustment. J. Gifted Educ. Creat. 10, 45–53.

[ref43] Silva F. Martorell M. (2024). La Batería de Socialización BAS3. Madrid: TEA.

[ref44] Smith M. M. Sherry S. B. Ge S. Y. Hewitt P. L. Flett G. L. Baggley D. L. (2022). Multidimensional perfectionism turns 30: a review of known knowns and known unknowns. Can. Psychol. 63, 16–31. doi: 10.1037/cap0000288

[ref45] Sternberg R. J. (2024). Individual, collective, and contextual aspects in the identification of giftedness. Gift. Educ. Int. 40, 3–24. doi: 10.1177/02614294231156986

[ref46] Stoeber J. (2018). “The psychology of perfectionism: an introduction” in The psychology of perfectionism: Theory, research, applications. ed. En Stoeber J. (London: Routledge/Taylor y Francis Group), 3–16.

[ref47] Stricker J. Buecker S. Schneider M. Preckel F. (2019). Multidimensional perfectionism and the big five personality traits: a Meta–analysis. Eur. J. Personal. 33, 176–196. doi: 10.1002/per.2186

[ref48] Turanzas JA Cordón JR Choca JP y Mestre JM (2020). Evaluación del programa APAC (mindfulness para superdotados) en una muestra española de niños superdotados: un estudio piloto. Mindfulness, 11, 86–98. doi: 10.1007/s12671-018-0985-1

[ref49] Vicent M. Inglés C. J. Gonzálvez C. Sanmartín R. Aparicio-Flores M. P. García-Fernández J. M. (2019). Perfectionism profiles and academic causal self-attributions in Spanish primary education students. Revista de Psicodidáctica 24, 103–110. doi: 10.1016/j.psicoe.2019.02.002

[ref50] Wang K. T. Rice K. G. Fu C. (2012). Perfectionism in gifted students: moderating effects of goal orientation and contingent self-worth. Sch. Psychol. Q. 27, 96–108. doi: 10.1037/a0029215, PMID: 22774784

[ref51] Yi S. Gentry M. (2021). Academic perfectionism of high-ability and high-achieving students in mathematics and science: differential relations by identification criteria of giftedness. Roeper Rev. 43, 173–186. doi: 10.1080/02783193.2021.1923592

[ref52] Zamudio S. C. Sierra M. D. L. D. V. Coronel G. E. O. Bravo J. F. F. Madrid I. M. (2024). “Alumnado con altas capacidades intelectuales. ¿Qué les preocupa a sus progenitores?” in Altas capacidades y educación: una aproximación desde la investigación (Madrid: Dykinson), 65–85.

[ref53] Zhao W. Wu A. M. S. Feng C. Yu K. Wang Z. Jiao K. (2024). Perfectionism and suicidal ideation: the serial mediating roles of appearance-based rejection sensitivity and loneliness. Curr. Psychol. 43, 25494–25503. doi: 10.1007/s12144-024-06230-y

