# Supplementary material for: Perfectionism and psychological well-being in adolescents with high intellectual abilities
Source: Front Psychol. 2025 Jul 22;16:1617755. doi: 10.3389/fpsyg.2025.1617755 (PMC12321751; doi:10.3389/fpsyg.2025.1617755)
Supplement: Supplementary file 1 [file Data_Sheet_1.pdf]

# FAMILY INTERVIEW

The data from this interview will only be used in the voluntary participation in the study by the University of Cádiz, for which consent was provided, and will be treated anonymously.

1. Email:
2. First and last name of your child:

**ADAPTATION**

3. Objective academic results your child is obtaining (check only one option, with an X)
   - Poor
   - Fair
   - Good
   - Excellent
4. Is the family atmosphere satisfactory/harmonious in relation to your child?

   ______________________________________________

   ______________________________________________

   ______________________________________________

5. Has your child been diagnosed with any psychological or mental health-related condition?

   ______________________________________________

6. How is your child's socialization? (meaning how do they relate to others)

   ______________________________________________

**FAMILY DATA**

7. Family socio-economic status (check only one option, with an X)
   - Low
   - Middle
   - High
8. Has there been any event that may have had a significant influence on your child's life? (check only one option, with an X)
   - Illnesses
   - Death of a family member
   - Absence of father or mother
   - Change of address

- Economic situation and unemployment in the family
- Divorce or separation
- None
- Other

9. Type of family your child lives in (check only one option, with an X)
   - Nuclear Family (mother, father, and children, typical classic family)
   - Extended Family (may include grandparents, aunts, uncles, cousins, and other relatives or close family friends)
   - Single-parent family (absolute absence of father or mother)
   - With separated or divorced parents (they fulfill their role as parents, although there is no couple)
   - Homoparental Family (formed by a same-sex couple and their children)
   - Reconstituted Family (separated people with children who form a new family unit)
   - Adoptive (who adopt children)
   - Other

10. Socioeconomic level and cultures of parents, mothers, or legal guardians (you can check several options, with an X)
    - Unemployment of one parent or caregiver
    - Unemployment of both parents or caregivers
    - Employed
    - Primary education
    - Secondary education
    - University education
    - Other

11. What people influence the education of your children? (you can check several options, with an X)

    - Father
    - Mother
    - Older siblings
    - Grandparents
    - Both legal guardians
    - Other (please specify)

12. Regarding your child's education, in general, do you, father, mother, or legal guardians, agree on what needs to be done? (check only one option, with an X)
    - Yes
    - No
    - It depends
13. How do you usually spend your free time as a family? (check only one option, with an X)
    - Individually
    - Together
14. The signs of affection you usually give your children are... (check only one option, with an X)

    - Frequent and direct
    - Infrequent and direct
    - Indirect
    - There are usually none
    - Other
15. When setting rules for your child's behaviour, do you usually explain the reasons? (check only one option, with an X)
    - Always
    - Sometimes
    - Never
16. Do you usually promise your children something that you later don't fulfill? (you can check more than one option, with an X)
    - No
    - Yes
    - I don't usually offer rewards for fulfilling their obligations
17. Does your child consider that the rules are established clearly and positively? (check only one option, with an X)
    - Yes
    - I don't know
    - No
    - Other

18. The most important thing in your child's education (check only one option, with an X)
    - Is that they learn to respect others
    - That they can develop their full potential
    - Is that they obey
19. The most important thing for your partner or ex-partner in your child's education (check only one option, with an X)
    - Is that they learn to respect others
    - That they can develop their full potential
    - Is that they obey
    - I don't have a partner or ex-partner
    - Other
20. In general, when a problem arises at home with my child, I think (I can choose several options, mark with X)
    - That I can solve it and I think of all the possible alternatives
    - Talk about it calmly most of the time
    - I think about the alternatives, but I almost always leave it to the last minute
    - I don't like to think about problems too much
21. When your child makes a mistake (check only one option, with an X)
    - You try to make them reflect on what happened
    - You usually punish them so they know they did wrong
    - You expect them to solve everything
22. In order for them to study, do you have to constantly force them or do they do it without being told anything? (check only one option, with an X)
    - You have to force them
    - They do it without being told anything
    - They don't do it
23. Does your child have a suitable place to study at home? (check only one option, with an X)
    - Yes
    - No
24. Does anyone help them with their studies? (you can check several options if applicable)
    - Mother

- Father
- Siblings
- Private tutor
- Academy
- They don't need help
- They don't tolerate help

25. Do they have a fixed study schedule?
    - Yes
    - No

**SCHOOL DATA**

26. Regarding your child's school performance, you are:
    - Satisfied
    - Normal
    - Not very satisfied
27. If you think they are underperforming, what do you think it could be due to? (you can check several options if applicable)
    - Due to capacity and individual differences
    - Due to study habits and parenting practices
    - Due to the methodology in the school setting
    - The atmosphere of coexistence in the school setting
    - Because parents see little of their children during the day, due to work
    - Due to divorce that may be affecting them
    - Other family circumstances that may be interfering
    - Other
28. What was your attitude towards the grades? (check only one option, with an X)
    - You agreed
    - You were indifferent
    - You considered them unfair
29. If they have siblings, in relation to them, your child is academically:
    - Better
    - The same
    - Worse

30. At home, is your children's school performance usually compared?
    - Always or almost always
    - Usually
    - Sometimes
    - Rarely
    - Never or almost never
    - Other
31. Do you think your child is comfortable at the institute? (check only one option, with an X)
    - Very much
    - Quite a bit
    - Normal
    - Little
    - Very little
    - Other
32. Do you think your child is satisfied in their class? (check only one option, with an X)
    - Very much
    - Quite a bit
    - Normal
    - Little
    - Very little
33. What type of profession or studies are you planning for your child?
    - University studies
    - Vocational training
    - That they work in the family business
    - School won't be worth much
    - The profession and/or studies they freely choose
34. What type of profession or studies do you think they will be able to pursue? (check only one option, with an X)
    - They will barely pass the ESO title if they achieve it
    - University studies
    - Vocational training
    - Profession that we can teach them in the family business
    - Right now, I don't see something they can project themselves into

- Other

**SOCIALIZATION**

35. Do they have friends? (check only one option, with an X)
    - Many
    - Some
    - Few
    - None
36. How do you think your child relates to their classmates? (check only one option, with an X)
    - Very well
    - Well
    - Fair
    - Poorly
37. Do you know your child's friends? (check only one option, with an X)
    - Yes
    - No
    - Some
38. Do you like your child to associate with them? (check only one option, with an X)
    - Yes
    - No
    - It depends

# ENTREVISTA FAMILIAR

Los datos de esta entrevista únicamente servirán en la participación voluntaria que realizan en el estudio de la Universidad de Cádiz, cuyo consentimiento facilitó y serán tratados de manera anónima.

1. **Correo electrónico: ______________________________________________________**

2. **Nombre y apellidos de su hijo/a:____________________________________________**

**ADAPTACIÓN**

3. **Resultados académicos objetivos** que viene obteniendo su hijo/a (marque sólo una opción, con una X)

**Malos___**
**Regulares___**
**Buenos____**
**Excelentes**____

4. ¿Es satisfactorio/armonioso el **clima familiar** y en relación a su hijo/a?

_________________________________________________________________________
_________________________________________________________________________
_________________________________________________________________________

5. ¿Presenta su hijo/a **algún diagnóstico de tipo psicológico o relacionado con la salud mental**?

_________________________________________________________________________

6. ¿Cómo es **la socialización** de su hijo/a? (quiere decirse cómo se relaciona con los demás)

_________________________________________________________________________

**DATOS FAMILIARES**

7. Situación socio-económica familiar (marque sólo una opción, con una X)
   - **Baja____**
   - **Media____**
   - **Alta**____

8. ¿Ha existido **algún acontecimiento que pueda haber ejercido influencia** importante en la vida de su hijo(a)? (marque sólo una opción, con una X)

**Enfermedades___**

**Muerte de un familiar____**
**Ausencia del padre o la madre____**
**Cambio de domicilio_____**
**Situación económica y desempleo en la familia____**
**Divorcio o separación_____**
**Ninguna_____**
**Otro_______________________________**

9. **Tipo de familia** en la que convive su hijo/a (marque sólo una opción, con una X)
   - **Familia Nuclear** (madre, padre y los hijos/as, típica familia clásica)_____
   - **Familia Extendida** (puede incluir abuelos, tíos, primos y otros consanguíneos o afines)_____
   - **Familia monoparental** (falta absoluta del padre o de la madre) _______
   - **Con padres separados o divorciados** (ejercen su papel como progenitores, aunque no hay pareja)______
   - **Familia Homoparental** (formada por una pareja del mismo sexo) y sus hijos/as)______
   - **Familia reconstituída** (personas separadas y con hijos/as que forman el nuevo núcleo familiar)_______
   - **Adoptiva** (que adoptan hijos/as) _______
   - **Otro______**

10. **Nivel Socioeconómico y culturas padres, madres o tutores legales** (puede indicar varias opciones, con una X)
    - **Desempleo de algún progenitor o cuidador______**
    - **Desempleo de ambos progenitores o cuidadores______**
    - **Activos laboralmente_____**
    - **Estudios primarios______**
    - **Estudios medios______**
    - **Estudios universitarios______**
    - **Otros______**

11. ¿Qué personas ejercen **influencia sobre la educación de sus hijos/as**? (puede marcar varias opciones, con una X)
    - **Padre_____**
    - **Madre______**
    - **Hermanos mayores______**
    - **Abuelos______**
    - **Ambos tutores legales_______**
    - **Otro (puede detallar la situación)___________________________________**

12. En cuanto a la educación de su hijo/a, de manera general piensen ¿**están de acuerdo, padre, madre o tutores legales**, en lo que hay que hacer? (marca sólo una opción, con una X)
    - **Sí____**
    - **No_____**

- **Depende_____**

13. Ustedes en familia ¿**Cómo suelen ocupar su tiempo libre**? (marca sólo una opción, con una X)
    - **De forma individual___**
    - **De forma conjunta___**

14. **Las muestras de cariño** que suelen darle a sus hijos/as, suelen ser…(marca sólo una opción, con una X)
    - **Frecuentes y directas_____**
    - **Infrecuentes y directas____**
    - **Indirectas_____**
    - **No suele haber____**
    - **Otro____**
15. Al establecer las normas para el comportamiento de tu hijo/a, ¿sueles explicarle las razones? (marca sólo una opción, con una X)
    - **Siempre___**
    - **A veces____**
    - **Nunca____**

16. **¿Le sueles prometer a tu hijo/as lo que después no cumples?** (puedes marcar más de una opción, con una X)

**No___**
**Sí____**
**No suelo ofrecerle recompensas por cumplir con sus obligaciones____**

17. Tu hijo ¿considera que **las normas están establecidas de manera clara y positiva?** (marca sólo una opción, con una X)

**Sí____**
**No lo sé_____**
**No_____**
**Otro_____**

18. **Lo más importante en la educación de tu hijo/a (**marca sólo una opción, con una X)

**Es que aprenda a respetar a los demás_____**
**Que pueda desarrollar todo su potencial______**
**Es que obedezca______**

19. **Lo más importante para tu pareja o expareja en la educación de tu hijo/a** (marcar sólo una opción, con una X)

**Es que aprenda a respetar a los demás_____**
**Que pueda desarrollar todo su potencial_____**
**Es que obedezca_____**
**No tengo pareja o expareja _____**
**Otro_____**

20. **En general, cuando surge un problema en casa con mi hijo/a, pienso (puedo elegir varias opciones, marcar con X )**
**Que podré resolverlo y pienso en todas las alternativas posibles____**
**Hablarlo desde la calma la mayor parte de las veces_____**
**Pienso en las alternativas, pero casi siempre lo dejo para el último momento_____**
**No me gusta demasiado pensar en los problemas_____**

21. **Cuando tu hijo/a comete un fallo (**marcar sólo una opción, con una X)
**Tratas de hacer reflexionar sobre lo ocurrido____**
**Sueles castigarle para que sepa que lo ha hecho mal____**
**Esperas que lo resuelva todo____**

22. **Para que estudie, ¿hay que forzarle constantemente o se pone sin decirle nada?** (marcar sólo una opción, con una X)
**Hay que forzarle____**
**Se pone sin decirle nada____**
**No se pone_____**

23. **¿Dispone su hijo/a en casa de un lugar adecuado para estudiar? (**marcar sólo una opción, con una X)
**Sí_______**
**No_______**

24. **¿Le ayuda alguien con el estudio?** (puede marcar varias opciones si así se tratara)

**Madre____**
**Padre_____**
**Hermanos_____**
**Profesor particular_____**
**Academia_____**
**No necesita ayuda_____**
**No tolera ayuda______**

25. **¿Tiene un horario fijo para estudiar?**
**Sí___**
**No__**

**DATOS ESCOLARES**

26. **Con el rendimiento escolar de su hijo/a están:**

**Satisfechos____**
**Normal______**
**Poco Satisfechos______**

27. **Si piensan que rinde por debajo de sus posibilidades, ¿a qué creen que puede deberse?** (puede marcar varias opciones si así se tratara)
**Por la capacidad y diferencias individuales____**

**Debido a los hábitos de estudio y prácticas de crianza_____**
**Debido a la metodología en el marco escolar_____**
**El ambiente de convivencia en el marco escolar______**
**Porque los padres vemos poco a los hijos durante el día, a causa del trabajo_____**
**Por divorcio que puede estar afectándole_____**
**Otras circunstancias familiares que pudieran estar interfiriendo_____**
**Otro____**

28. **¿Cuál fue la actitud de ustedes ante las calificaciones? (**marcar sólo una opción, con una X)
    **Estuvieron de acuerdo_______**
    **Se mostraron indiferentes______**
    **Las consideraron injustas______**

29. **Si tiene hermanos/as y en relación a ellos, su hijo/a, escolarmente resulta:**
    **Mejor___**

    **Igual____**

    **Peor____**

30. **En casa, ¿se suele comparar el rendimiento escolar de sus hijos/as?**
    **Siempre o casi siempre____**
    **Habitualmente___**
    **A veces____**
    **Pocas veces_____**
    **Nunca o casi nunca____**
    **Otro____**

31. **¿Creen que su hijo/a se encuentra a gusto en el instituto? (**marcar sólo una opción, con una X)
    **Mucho____**
    **Bastante____**
    **Normal_____**
    **Poco_____**
    **Muy poco_____**
    **Otro____**

32. **¿Creen que su hijo/a se encuentra satisfecho en su clase?** (marcar sólo una opción, con una X)
    **Mucho___**
    **Bastante____**
    **Normal_____**
    **Poco_____**
    **Muy poco____**

33. **¿Qué tipo de profesión o estudios tiene pensado para su hijo?**
    **Estudios Universitarios___**

**Formación profesional____**
**Que trabaje en la empresa o negocio familiar____**
**El colegio no le va a valer mucho____**
**La profesión y/o estudios que elija libremente____**

34. **¿Qué tipo de profesión o estudios creen que va a poder hacer?** (marcar sólo una opción, con una X)
**Sacarse con dificultad si lo consigue el título de la ESO____**
**Universitarios____**
**Formación profesional___**
**Profesión que podamos enseñarle en el negocio familiar____**
**Ahora mismo, no veo algo en lo que pueda proyectarse____**
**Otro____**

**SOCIALIZACIÓN**

35. **¿Tiene amigos?** (marcar sólo una opción, con una X)
**Muchos____**
**Algunos____**
**Pocos_____**
**Ninguno_____**

36. **¿Cómo creen que su hijo/a se relaciona con sus compañeros/as?** (marcar sólo una opción, con una X)
**Muy Bien____**
**Bien_____**
**Regular_____**
**Mal____**

37. **¿Conoce a los amigos de su hijo/a?** (marcar sólo una opción, con una X)
**Sí____**
**No___**
**Algunos____**

38. **¿Les gusta que su hijo/a se relacione con ellos/as?** (marcar sólo una opción, con una X)
**Sí___**
**No___**
**Depende____**
